# Air change rate vs airflow pathway: bioaerosol containment and removal in patient rooms

**DOI:** 10.1186/2047-2994-4-S1-P94

**Published:** 2015-06-16

**Authors:** K Grosskopf

**Affiliations:** 1 Engineering, University of Nebraska, Lincoln, NE, USA

## Introduction

Recent studies have shown that higher air change rates may have the unintended consequence of creating turbulent airflows that entrain high concentrations of infectious particles within the breathing zone, and possibly, breakdown pressure relationships necessary to contain the spread of infectious particles to other clinical spaces.

## Objectives

A series of experimental and numerical tests were conducted in an actual hospital to observe the containment and removal of respirable aerosols (0.5-10µm) with respect to ventilation rate and directional airflow in a general patient room, and, an airborne infectious isolation room (AIIR).

## Methods

A total of four experimental tests were conducted; two each in a general patient room and an infectious isolation room. A synthetic oil (polyaliphatic olefin) was continuously aerosolized at a rate of 15mg/0.4L of air per second to generate an aerosol (0.5µm - 10µm) at the approximate height of a patient lying at rest (0.8m). Particle size distribution samples were drawn at 30 sec intervals at 30 sampling locations in the test rooms over 4 hours. Computational analyses were used to validate the experimental results, and, to further quantify the particle transport phenomena.

## Results

Increasing mechanical ventilation from 2.5 to 5.5 ACH reduced aerosol concentrations only 30% on average. However, particle concentrations were more than 40% higher in pathways between the source and exhaust as was the suspension and migration of larger particles (3 - 10µm) throughout the patient room(s). Higher ventilation rates did not appear to affect directional airflow relationships between corridors and patient rooms having anterooms and a pressure differential of ≥ 2.5Pa.

## Conclusion

Higher ventilation rates were not found to be *proportionately* effective in reducing aerosol concentrations. Airflow pathways, not air change rates, were found to be the dominant environmental factor for bioaerosol migration and potential cross-infection.

## Disclosure of interest

None declared.
